# SegDecon bridges histology and transcriptomics through AI-based nuclei segmentation and image-informed spatial deconvolution

**DOI:** 10.1016/j.csbj.2025.10.041

**Published:** 2025-10-24

**Authors:** Yuesi Xi, Xun Jiang, Jonas C. Schupp, Cheng-Jian Xu, Yang Li

**Affiliations:** a Centre for Individualised Infection Medicine (CiiM), a joint venture between the Helmholtz-Centre for Infection Research (HZI) and the Hannover Medical School (MHH), Hannover, Germany; b Twincore, a joint venture between the HZI and the MHH, Hannover, Germany; c Lower Saxony Center for Artificial Intelligence and Causal Methods in Medicine (CAIMed), Hannover, Germany; d Department of Respiratory Medicine, Hannover Medical School, Hannover, Germany; e Biomedical Research in End-Stage and Obstructive Lung Disease (BREATH), Hannover Medical School (MHH), German Center for Lung Research (DZL), Hannover, Germany; f Department of Clinical Airway Research, Fraunhofer Institute for Toxicology and Experimental Medicine (ITEM), Hannover, Germany; g Pulmonary, Critical Care, and Sleep Medicine, Yale University, New Haven, USA; h Department of Internal Medicine and Radboud Center for Infectious Diseases, Radboud University Medical Center, Nijmegen, the Netherlands; i Cluster of excellence Resolving Infection Susceptibility (RESIST, EXC 2155), Hannover Medical School, Hannover, Germany; j Biomedical Research in Endstage and Obstructive Lung Disease Hannover (BREATH), German Center for Lung Research (DZL), Hannover, Germany

**Keywords:** Spatial transcriptomics, Segmentation, Deconvolution, Histology

## Abstract

Precise spatial mapping of cellular composition is a central goal in spatial transcriptomics (ST), yet current methods often assume uniform or manually estimated cell counts across spatial spots, potentially distorting biological interpretation. Here, we present SegDecon, a computational framework that integrates image-derived cell count estimation into Bayesian deconvolution. SegDecon enhances nuclei segmentation using Hue-Saturation-Value (HSV) color space transformation, morphological filtering, and deep learning-based instance segmentation. It quantifies nuclei per spatial spot and refines cell-type deconvolution through tailored Gamma priors in a modified cell2location model. Evaluated on high-resolution mouse brain ST data, SegDecon demonstrates improved correlation with ground truth, particularly in resolving low-abundance and spatially restricted cell types. This approach provides a reproducible and accessible solution to bridge histology with transcriptomic deconvolution, improving both resolution and biological fidelity. Source code is available at: https://github.com/CiiM-Bioinformatics-group/SegDecon

## 1 Introduction

Spatial transcriptomics enables spatially resolved gene-expression profiling across intact tissue sections, providing insights into cellular organization and tissue architecture. In widely used platforms such as 10x Genomics Visium, the effective resolution (∼50–100 µm per capture spot, ∼55 µm diameter) means that each spot typically aggregates multiple cells. Consequently, computational deconvolution is required to infer spot-level cell-type composition using single-cell RNA-seq references. However, many popular approaches, including cell2location [1], RCTD [2], and Stereoscope [3] employ fixed or heuristically defined priors for the number of cells per spot, which can misrepresent genuine spatial variability in local cellularity and bias downstream estimates.

Hematoxylin and Eosin (H&E)-stained tissue images provide morphological context that can inform local cellularity [4]. Yet robust nuclei segmentation remains challenging: low contrast, uneven staining, noisy backgrounds, and overlapping or irregular nuclei frequently degrade conventional methods [5], [6], [7]. In standard-resolution Visium, such instability weakens image-informed priors and, in turn, reduces deconvolution accuracy. Despite the promise of histology for estimating spot-level cell density, few current ST deconvolution tools explicitly incorporate spot-level morphological information into their models. While deconvolution is unnecessary for single-cell-scale platforms (e.g., Visium HD, ∼2 µm bins), accurate nuclei segmentation is still required to assemble subcellular bins into cell-level units and to register transcript counts to individual cells (e.g., StarDist-based workflows) [8], [9], [10], [11]. Accordingly, the same segmentation improvements remain valuable for HD data, an ancillary benefit rather than the focus of this work.

**Fig. 1:**
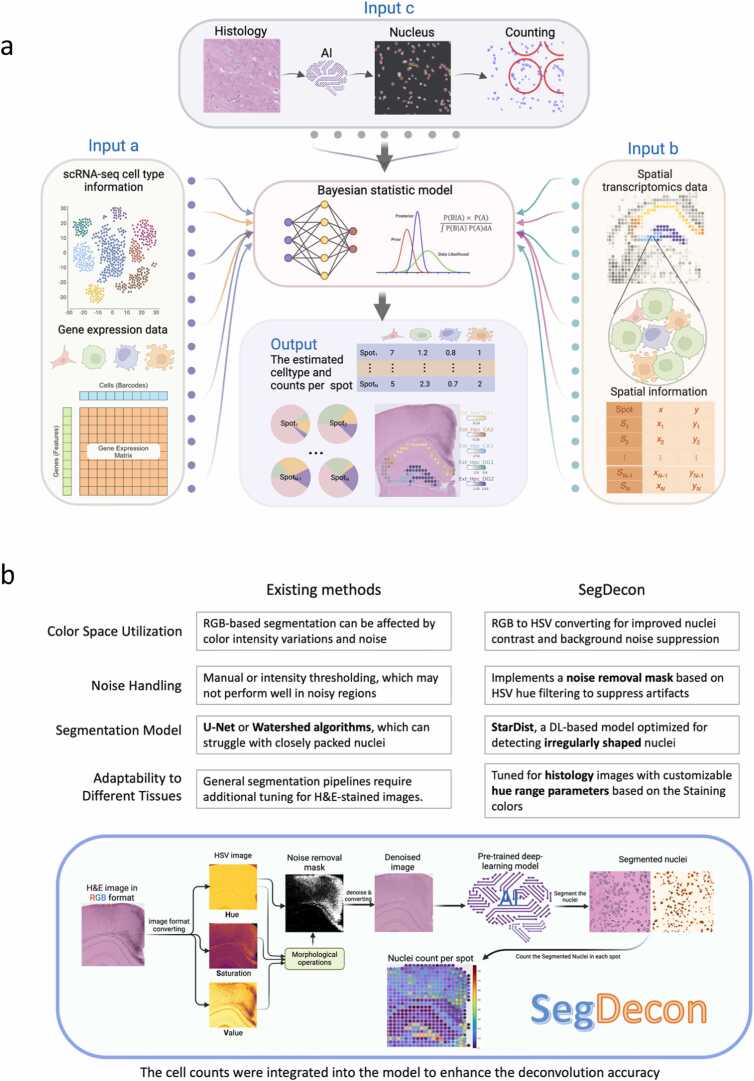
SegDecon and Nuclei segmentation framework **(a)** A schematic overview of the SegDecon framework, which integrates three distinct types of inputs for ST deconvolution. Input a represents scRNA-seq data with cell-type-specific gene expression profiles, serving as a reference to map ST data to known cell types. Input b consists of spatial transcriptomics data, including spot-level gene expression matrices and spatial metadata, ensuring that inferred cell compositions align with the tissue's spatial organization. Input c involves histological data derived from H&E-stained images, where AI-driven segmentation provides image-derived nuclei counts information for each spatial spot, addressing variability in cell density. These inputs are integrated into a Bayesian statistical model to estimate cell-type proportions and absolute cell counts per spot. The outputs include spatially resolved cell-type distributions and tissue maps. **(b)**This figure compares SegDecon with traditional segmentation approaches and illustrates its workflow. SegDecon enhances nuclei detection by converting RGB images to HSV for improved contrast and noise suppression. It applies a hue-based noise removal mask and uses StarDist, a deep-learning model optimized for irregularly shaped nuclei.

To address these needs, we present SegDecon, a pipeline that bridges histology and transcriptomics by coupling histology-guided cell-count estimation with Bayesian deconvolution model (Fig. 1a). On the histology image side, SegDecon enhances nuclei detection via Hue-Saturation-Value (HSV) color-space transformation with hue-based noise masking and morphology-aware filtering, followed by StarDist instance segmentation, yielding stable spot-level, image-derived nuclei counts (Fig. 1b). On the modeling side, these counts are integrated into a modified cell2location [1] framework by replacing fixed priors on total cell abundance with a Gamma prior on each spot’s total abundance with shared hyperparameters (κ,θ) estimated by moment-matching to the global (or ROI-specific) nuclei-count moments of these image-derived counts. This data-adaptive design adjusts prior strength to local heterogeneity and improves the accuracy of both total cell numbers and cell-type proportions across diverse tissue regions. We also provide training and evaluation scripts to facilitate reproducible use.

Relation to prior work and positioning. Recent work has begun to incorporate histology into spatial deconvolution (e.g., Spotiphy [12], which jointly models nuclei counts and gene expression). SegDecon differs in both emphasis and implementation: (i) an explicit per-spot empirical prior (κ, θ) within a modified bayesian deconvolution framework, and (ii) a transparent, deployment-friendly segmentation stack that improves counts before modeling. We later assess SegDecon alongside related methods under matched datasets and metrics (Results) and discuss methodological implications and limits in the Discussion.

## 2 Materials and methods

### 2.1 Dataset acquisition and preprocessing datasets

We analyzed two cohorts: (i) an internal high-resolution cohort comprising 10x Genomics Visium HD mouse brain data from which we synthesized standard-resolution pseudo-spots (“meta-Visium”), and (ii) an external cohort comprising native 10x Genomics Visium mouse brain data. All histology images were H&E-stained. Raw count matrices and images were processed with Scanpy v1.10.3 and stored as AnnData objects [13]; vendor scale factors and spatial coordinates were preserved to maintain geometric fidelity.

### 2.2 Internal cohort (Visium HD → meta-Visium)

Visium HD (FFPE, Mouse Brain) provides whole-transcriptome measurements on ∼2 µm capture bins [14]. To emulate standard Visium geometry, we generated a hexagonal grid with 55 µm spot diameter and ∼100 µm center-to-center spacing. For each pseudo-spot with center c_s, we summed raw UMI counts from all HD bins whose centers lie within 27.5 µm of $c_{s}$ (Euclidean radius search via scipy.spatial.cKDTree [15]). Aggregation was purely geometric on raw transcript counts; no nuclei/cell labels or histology-derived annotations were used to construct pseudo-spots. We produced two separate AnnData objects: (i) an HD bin-level object (bin coordinates and raw counts), and (ii) a meta-Visium spot-level object (pseudo-spot coordinates and aggregated counts). Both objects preserve vendor scale factors and spatial transforms. Deconvolution used only the meta-Visium (pseudo-spot) AnnData, while the HD bin-level AnnData was used exclusively as a high-resolution reference for an unbiased evaluation (with no information leakage).

### 2.3 External cohort (native Visium)

For external evaluation at true Visium resolution, we used the CytAssist-Preservation Method Comparison on CytAssist: Fresh Frozen Mouse Brain (Sagittal), 11 mm Capture Area dataset (CytAssist_Fresh_Frozen_Sagittal_Mouse_Brain) [16]. Vendor-provided spot layouts, tissue masks, and scale factors were used to register spots to the histology frame. Raw UMI count matrices and spatial metadata were ingested without modification into AnnData. All analyses on this cohort were performed at native Visium resolution.

### 2.4 Preprocessing and quality control

Unless otherwise noted, raw UMI counts were used for deconvolution. Library-size normalization and log1p transforms were applied only for exploratory visualization (e.g., QC plots). No smoothing or imputation was applied prior to deconvolution. Standard QC filters (e.g., minimum UMIs per spot and mitochondrial content thresholds) were applied uniformly across cohorts.

## 3 Histology Image Processing and Nuclei Segmentation

H&E histology images were converted from RGB to HSV using OpenCV (cv2.cvtColor) [17]. In HSV space, hue provides chromatic contrast less sensitive to intensity drift, improving nuclei-background separability. A tissue mask was generated by default from the HSV saturation (S) channel, or optionally from the hematoxylin channel after RGB→ Hematoxylin-Eosin-DAB conversion (scikit-image color.rgb2hed) [18], [19], followed by Otsu thresholding; small components and holes were removed with a resolution-aware area cutoff, and a disk-like closing standardized tissue boundaries (defaults in Supplementary Methods, Parameter table).

To suppress stain-like artifacts during preprocessing, we constructed a hue-based artifact mask within the tissue region. Hue values were percentile-clipped (default 5th-95th) and normalized; candidate thresholds (Yen, Triangle, Otsu) considering both high- and low-hue tails, were screened to achieve a target artifact-area fraction (default 0.5–30 % of tissue) [20], [21], [22]. If no candidate satisfied the target, extreme quantiles were used, selecting the side with smaller area. Tiny components were removed via a size threshold proportional to the expected nuclear area; small holes were filled.

Background homogenization was then applied only to non-nuclear tissue. An S-weighted circular mean defined a “pink core”; pixels outside a narrow circular-hue band (width q=0.30) and outside robust saturation/value(S/V) ranges defined by median absolute deviation (MAD [23]; a robust spread estimator computed as median|X−median(X)|, using the normal-consistent version**)** with multipliers $k_{s}=k_{v}=2.0$) were replaced by the core median HSV and lightly bilateral-filtered (filtering restricted to replaced pixels). This reduces haloing and low-frequency background while preserving nuclear chroma and edges.

Preprocessed images were submitted to StarDist (pretrained 2D model) for instance segmentation into star-convex polygons [8], [9], [10]. Regions flagged by the artifact mask were set to a uniform background prior to inference. Post hoc, nuclei with area in (50, 2000) µm² were retained; surviving polygons and centroids were exported for downstream mapping. Full defaults and implementation details are provided in Supplementary Methods.

## 4 Segmentation benchmarking: ROI selection, tiling, and noise scoring

For each cohort (internal Visium HD-FFPE mouse brain; external CytAssist fresh-frozen sagittal mouse brain), we identified a 3000 × 3000 px ROI with high artifact burden while maintaining adequate tissue coverage, using the binary artifact mask above. Each ROI was partitioned into a 6 × 6 grid of 500 × 500 px tiles (36 tiles per cohort; 72 total). We computed a HASS per tile (artifact area fraction, 95th-percentile component size, edge-overlap with V-channel Canny, blue-nuclei proximity, and local texture drop). Tiles were stratified into five noise levels (1–5; higher = worse). All segmentation methods were evaluated on exactly the same tiles; metrics were computed per tile and aggregated by cohort. Formal HASS definitions and ROI/tile coordinates are provided in the Supplementary Methods.

## 5 Manual ground-truth (GT) nuclei counts and evaluation metricsh

To obtain a reference for tile-wise counting accuracy, we created manual ground truth (GT) by annotating nuclei centroids on the same artifact-enriched ROIs and 6 × 6 tile grids described above (36 tiles; 500 × 500 px each). For each tile, annotators clicked visible nuclei centers on the H&E image; the interface recorded both tile-local and full-image coordinates and wrote per-tile GT counts (units: nuclei/tile). GT is used only for count-level evaluation and not as a polygonal mask or for model fitting.

Predicted nuclei (same StarDist model and post-hoc small area filter) were mapped to tiles under the common rule described in *Segmentation benchmarking*. We then computed absolute counting error |pred−gt| (nuclei/tile) and Bland-Altman agreement statistics (mean bias; 95 % limits of agreement). Significance was assessed with paired Wilcoxon tests with Holm adjustment. Throughout the Results, gt denotes these manual GT counts. Annotation scripts and commands are provided in the Supplementary Methods — Code Listings and Evaluation Protocols (Listings S1-S8).

## 6 Preprocessing ablation setup

A controlled preprocessing ablation quantified component-wise effects on downstream nuclei segmentation and tile-level counting. A single baseline (PP01_base) and four single-change ablations (AB01-AB04) were executed deterministically (NumPy seed = 0) on the same H&E slide/ROI. Images were loaded as 8-bit RGB and processed at 0.5 µm/px with an expected nuclear diameter of 10 µm; an odd, resolution-aware kernel size was derived from this scale.

### 6.1 Baseline (PP01_base)

(i) tissue mask from HSV-S (Otsu; area/hole filtering; elliptical closing); (ii) hue-based artifact mask within tissue after 5–95 % clipping, selecting Yen/Triangle/Otsu thresholds with a target area of 0.5–30 % (quantile fallback if unmet); (iii) removal of tiny components < 0.35 × π(d/2)² with hole filling; (iv) “keep-nuclei + pink replacement” without black-hat: preserve nuclei, define a pink band by circular-hue quantile width (q_width = 0.30) plus MAD constraints on S and V (k_s = k_v = 2.0), replace non-pink, non-nuclear tissue with median pink HSV, and lightly smooth the replaced region.

### 6.2 Ablations

AB01_disable_MAD (remove S/V MAD constraints); AB02_force_quantile (force quantile fallback for the hue mask); AB03_no_smallfilter (disable tiny-component filtering and hole filling); AB04_dilate2 (dilate the nuclei mask by 2 px before replacement). Preprocessed images were percentile-normalized (5–95 %) and segmented with StarDist 2D (Versatile HE) using predict_instances_big (block_size = 4096, min_overlap = 128, context = 128, n_tiles = (4,4,1), prob_thresh = 0.2). StarDist polygons were filtered by area and assigned to ROI tiles by centroid; manual point annotations were aggregated per tile as ground truth. Metrics included ME, MAE, RMSE, and Bland-Altman mean difference with 95 % limits of agreement. Consolidated results are reported in Supplementary Table S7 (Table_S7_ablations_AB01_AB04.csv).

## 7 Spatial mapping and nuclei counting

Histology images were registered to spatial barcodes using vendor scale factors. Although Visium spots are physically 55 µm in diameter with 100 µm center-to-center spacing, prior reports of spot swapping/bleed and the physics of permeabilization indicate that capture reflects a proximal neighborhood rather than a perfectly sharp footprint [24], [25]; accordingly, we modeled each spot as a disc with an effective capture radius of 45 µm (discs remain non-overlapping at 100 µm pitch). This choice does not modify transcript counts (which are used exactly as provided by Space Ranger); it only affects the image-derived nuclei counts used to construct the empirical prior. Segmented nuclei (StarDist polygons) were mapped to spots by geometric intersection with the 45 µm discs (intersection area > 0; ties broken by larger overlap fraction). Nuclei with area outside (50, 2000) µm² were excluded. The resulting per-spot nucleus count $n_{s}$ served as the image-derived estimate of local cellularity.

Across spots S, we computed

$$\overset{®}{n}=\frac{1}{|S|}\sum_{s\in S}n_{s},\;\sigma_{n}^{2}=\frac{1}{\left|S\right|-1}\sum_{s\in S}{\left(n_{s}-\overset{®}{n}\right)}^{2},\mathrm{VMR}=\frac{\sigma_{n}^{2}}{\overset{®}{n}}$$

These moments parameterize the Gamma prior in the deconvolution model (next section).

### 7.1 Bayesian deconvolution model with empirical priors

We modify the cell2location framework to incorporate image-derived nuclei counts as priors on the spot-level total cell abundance. For gene g at spot s, we model observed counts $y_{\mathrm{gs}}$ with a negative binomial (NB) distribution parameterized by the mean $\mu_{\mathrm{gs}}$ and a gene-specific inverse- dispersion $\alpha_{g}$(mean-size/NB2 parameterization; $\alpha_{g}$is gene specific inverse-dispersion):

$$y_{\mathrm{gs}}∼\mathrm{NB}\left(\mu_{\mathrm{gs}},\alpha_{g}\right),\mathrm{Var}\left(y_{\mathrm{gs}}|,\mu_{\mathrm{gs}},\alpha_{g}\right)=\;\mu_{\mathrm{gs}}+\frac{\mu_{\mathrm{gs}}^{2}}{\alpha_{g}}\;$$

The expected expression factorizes into a spot-level total abundance and a reference-weighted cell-type mixture:

$${\mu_{\mathrm{gs}}=m}_{s}∙\sum_{k}w_{\mathrm{ks}}r_{\mathrm{gk}}$$

with $m_{s}$ is the total cell abundance at spot s; $w_{\mathrm{ks}}\geq 0$ with $\sum_{k}w_{\mathrm{ks}}$ = 1 are cell-type proportions; $r_{\mathrm{gk}}$ denotes the reference expression of the gene g in cell type k from the scRNA-seq atlas.

To incorporate information from histology, we place a Gamma prior on $m_{s}$, in shape-rate form:

$$m_{s}∼\mathrm{Gamma}\left(\kappa ,\theta \right),Ε\left[m_{s}\right]=\;\frac{\kappa }{\theta }\;,\mathrm{Var}\left[m_{s}\right]=\;\frac{\kappa }{\theta^{2}}$$

Let S denote the set of all spatial spots retained for analysis, and let |S| be its cardinality. For each spot s∈S, let $n_{s}$ be the nucleus count obtained from the image pipeline. We compute the sample mean and unbiased sample variance across all spots,

$$\bar{n}=\;\frac{1}{|S|}\sum_{s\in S}n_{s},\;\sigma_{n}^{2}=\frac{1}{\left|S\right|-1}\sum_{s\epsilon S}{\left(n_{s}-\bar{n}\right)}^{2}$$

and define the variance-to-mean ratio $\mathrm{VMR}=\sigma_{n}^{2}/\overset{®}{n}\;$(index of dispersion).

where:

$n_{s}$ is the integer nuclei count at spot s (units: nuclei/spot);

S is the set of analyzed spots; |S| is the number of such spots;

$\bar{n}$ is the sample mean nuclei count; $\sigma_{n}^{2}$ is the sample variance.

By moment matching the Gamma prior on $m_{s}$ (shape-rate form) to these empirical moments, we obtain the closed-form hyperparameters

$$\kappa =\frac{{\bar{n}}^{2}}{\sigma_{n}^{2}}=\frac{\bar{n}}{\mathrm{VMR}},\theta =\frac{\bar{n}}{\sigma_{n}^{2}}=\frac{1}{\mathrm{VMR}}$$

So that smaller VMR implies a tighter prior (larger κ); larger VMR yields a weaker prior.

Notation. $\alpha_{g}$is reserved for NB inverse-dispersion; (κ,θ)denote Gamma shape and rate; $\mu_{\mathrm{gs}}$ is the NB mean for (g,s).

## 8 Cell-Type Composition and Absolute Count Estimation

After posterior inference, we report cell-type proportions $w_{k,s}$ per spot directly. To obtain absolute cell-type counts, we multiply these proportions by the image-derived nucleus count of the same spot:

$$C_{k,s}=w_{\mathrm{ks}}∙n_{s}$$

where $n_{s}$ is the per-spot nucleus count computed from nuclei segmentation and spatial mapping (see Spatial Mapping and Nuclei Counting). This post hoc scaling yields an interpretable estimate of cell-type abundance without altering transcript counts (which remain as provided by Space Ranger). Note that $n_{s}$ are also summarized globally (via $\bar{n},\sigma^{2}$, and $\mathrm{VMR}=\sigma^{2}/\;\bar{n}$) solely to set the shared Gamma prior Gamma(κ,θ)for $m_{s}$; per-spot $n_{s}$values are not fed back into the model likelihood.

## 9 Parameter optimization and threshold selection

All thresholds and kernels in the histology pipeline are exposed as resolution-aware parameters. Unless otherwise stated, the default configuration was used for all external validations (no per-dataset tuning): acquisition scale μm_per_px = 0.5; expected nucleus diameter = 10μm; tissue masking via the S channel (or the hematoxylin component from RGB→HED) with a minimum tissue-object area of 400 µm² and a closing radius proportional to the expected nuclear size; hue-artifact suppression with percentile clipping (5, 95) and a target artifact-area fraction of 0.5–30 %; tiny-component removal at 0.35 × the expected nuclear area (optionally overridden by an absolute area in µm²); background homogenization with q_width=0.30, $k_{S}=\;k_{V}=\;2.0$, optional nucleus-safety dilation 0–3 px (default 0); StarDist pretrained 2D model; and a post-hoc area filter of (50, 2000) µm². Practical presets for atypical slides, along with a complete CLI parameter map (names, defaults, ranges, roles), are provided in Supplementary Methods (Parameter table: defaults and roles).

## 10 Computing environment and resource usage

All experiments used the default configuration (no per-dataset tuning) and were run on a workstation with a single NVIDIA GPU (Tesla 4, 16 GB) and 128 GB RAM. SegDecon histology preprocessing is CPU-only and completes within 5 min per whole-slide image (high-resolution H&E). StarDist [8], [9], [10] segmentation and cell2location [1] deconvolution were executed on the GPU; wall-clock time scales with spot count and gene panel size and is on the order of minutes per slide. Full environment details and exact commands are provided in the Supplementary Methods (Runtime, software environment, and seeds).

## 11 HD cell reconstruction, QC, and annotation (reference only)

For Visium HD, we reconstructed cell-level expression profiles by mapping HD capture bins (∼2 µm) to instance-segmented nuclei via geometric overlap. Each HD bin was assigned to the nucleus whose mask contained the bin centroid (ties broken by maximal area overlap). Per-nucleus UMI count vectors were obtained by summing the counts of assigned bins; the nucleus centroid was retained as its spatial coordinate. These data were stored as a dedicated AnnData object.

Quality control. After assigning HD bins to nuclei, we retained nuclei whose mask area lay strictly between 50 and 2000 µm² (i.e., area > 50 and < 2000), removing very small debris and likely merged objects. We additionally required per-nucleus total UMI counts > 50 (from the total_counts column). These filters were applied sequentially (area range, then UMI threshold). No smoothing or imputation was applied prior to labeling.

Cell-type annotation. Marker genes were curated from matched scRNA-seq references [1], [26], [27]. We computed per-nucleus enrichment scores using decoupleR (v1.8.0) [26] and transferred labels by assigning the top-scoring cell type per nucleus; ties were resolved by preference to lineage-consistent categories. These annotations were used only for evaluation and visualization of HD-derived references and were not used during deconvolution on meta-Visium pseudo-spots (to avoid information leakage).

## 12 Results

### 12.1 Rationale and development of SegDecon

Accurate spatial deconvolution depends critically on reliable cell count estimation from histology and well-specified priors on local cellularity, yet both remain major bottlenecks in current spatial transcriptomics workflows. Conventional H&E-based segmentation often fails under variable staining and background noise, leading to unstable spot-level nuclei counts, while deconvolution models typically assume uniform or heuristic priors on total cell abundance, overlooking true spatial variation in tissue density. These limitations reduce biological fidelity and hinder quantitative comparison across regions or datasets.

To overcome these challenges, we developed SegDecon, a pipeline that couples HSV-guided nuclei segmentation with image-informed Bayesian deconvolution (Fig. 1a, b). The core idea is to extract robust, image-derived estimates of cellularity directly from histology and use them to calibrate data-adaptive Gamma priors on total abundance within a modified cell2location framework anchoring the model to morphological evidence without altering transcript counts or likelihoods.

We evaluated SegDecon using mouse brain snRNA-seq and 10x Visium HD spatial datasets. Cross-modal annotation was consistent (59 cell types; related subtypes grouped), providing a reliable foundation for benchmarking (Fig. S1a, b). To enable controlled testing at conventional Visium scales, we aggregated HD capture bins into ∼55 µm pseudo-spots, preserving spatial geometry and generating Visium-like data with known structure (Fig. S1c, d).

### 12.2 HSV improves nuclei-segmentation accuracy and robustness

**Fig. 2:**
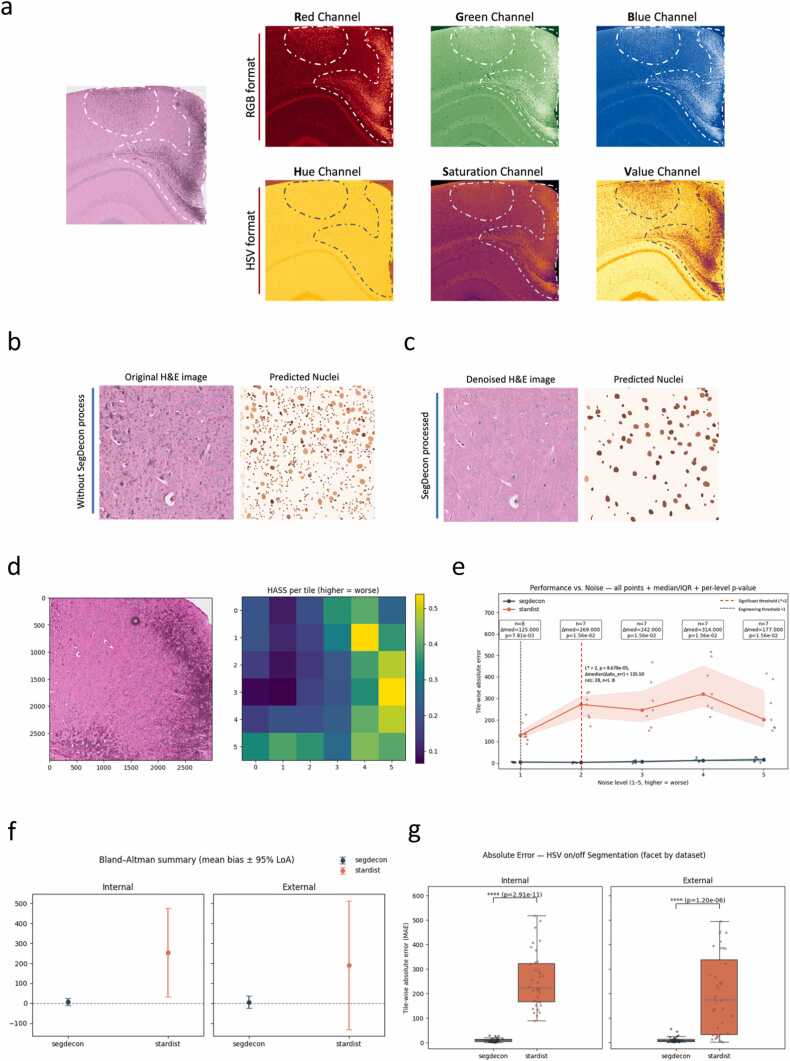
Performance of the nuclei segmentation (a)Microscopy image of H&E-stained mouse brain in RGB and HSV formats. The top row shows the red, green, and blue channels of the RGB format, where the cropped regions exhibit strong background noise across all channels. The bottom row presents the hue, saturation, and value channels of the HSV format. The hue channel effectively suppresses background noise and highlights the nuclei with enhanced contrast. (b)Segmentation of the nuclei without SegDecon preprocessing: original H&E (left) and predicted nuclei (right).(c)Segmentation of the nuclei with SegDecon preprocessing: original H&E (left) and predicted nuclei (right).(d)Artifact heatmap of HASS on a 3000 × 3000 px crop (Data1). The slide is partitioned into a 6 × 6 grid; each tile receives an H&E HASS combining five interpretable terms derived from a hue-based speckle mask: speckle area fraction, 95th-percentile connected-component size (P95_norm), edge-overlap index with V-channel edges, blue/purple-nuclei proximity and local texture-degradation index. Tiles are binned into five levels by within-slide HASS quintiles (1–5; higher = worse). (e)Performance *vs.* noise (tile-wise absolute counting error). StarDist (baseline) against SegDecon (HSV denoising + StarDist) across HASS levels 1–5. Points are tiles (with horizontal jitter per method); lines trace the per-level median; shading indicates IQR. Labels above bins show paired Wilcoxon tests (n = paired tiles; Δmedian = median (|err|_StarDist_ − |err|_*SegDecon*_); two-sided p). The dashed red line marks the significant threshold L* obtained by comparing Δ|err| in tiles with noise ≥ L vs. < L (Mann-Whitney U); here L*= 2 (p = 8.68 ×10⁻⁵; Δmedian=135.5; n_≥L=28, n < L=8). A gray dotted line marks an engineering threshold L= 1 (criterion Δ|err|>5). (f)Bland-Altman summary (mean bias ±95 % limits of agreement) for HSV on/off, faceted by dataset (Internal/External). Dots show mean bias; bars indicate LoA. (g)Absolute error with HSV on vs. off, by dataset. Boxplots show median and IQR; whiskers = 1.5 ×IQR; jittered points are tiles. Paired Wilcoxon p-values with Holm adjustment are annotated.

A central obstacle lies in achieving stable nuclei segmentation under uneven staining and low contrast. Converting H&E images from RGB to HSV isolates chromatic information in the hue channel, effectively separating nuclei from background (Fig. 2a). Combined with StarDist [8], [9], [10], this produced accurate, reproducible segmentation with improved morphology and separation (Fig. 2b, c). Across both internal Visium HD-FFPE and external CytAssist fresh-frozen slides, HSV preprocessing reduced tile-wise absolute counting error and centered Bland–Altman bias near zero with tighter 95 % limits of agreement (Fig. 2f, e). A per-tile H&E Artifact Severity Score (HASS) summarized staining heterogeneity (Fig. 2d); stratification by HASS showed uniformly lower errors for SegDecon compared to StarDist-only, with pronounced gains at higher artifact levels (L* = 2, Δmedian |err| = 135.5 nuclei/tile, p = 8.68 × 10⁻⁵) and consistent improvements without dataset-specific tuning (Fig. 2e).

At the spatial level, nuclei count per spot closely recapitulated known anatomy (Fig. S1e, f) and revealed regions where expression-only estimates diverged from true cellularity (compare Fig. S1c, f), underscoring the value of image-informed calibration. These empirical nuclei count form the foundation for SegDecon’s adaptive Gamma priors, improving downstream alignment between predicted and annotated cell-type distributions while maintaining full model interpretability.

### 12.3 Module-wise ablations demonstrate overall robustness of the preprocessing stack

To isolate the contribution of each preprocessing step and stress-test robustness, we performed module-wise ablations, starting from the default stack and toggling one component at a time. Among these single-step changes with the default configuration, removing the small-object filter (AB03) produced the largest and most consistent degradation in counting accuracy (internal p = 2.91 × 10⁻¹¹; external p = 1.08 × 10⁻⁸) (Fig. S3a). Heatmaps corroborated this pattern (substantially positive ΔMAE with ΔR² < 0 for AB03), whereas forcing the quantile fallback (AB02) had negligible impact internally but yielded a modest improvement externally (p = 1.52 × 10⁻²) (Fig. S3b). Expanding the nucleus-safety dilation to 2 px (AB04) caused a mild regression, and disabling S/V MAD constraints (AB01) was effectively neutral across slides (Fig. S3a-b). Under the default stack, per-tile error distributions remained well centered with narrow spread, underscoring overall robustness (Fig. S3c). Applying the same HSV preprocessing upstream of Spotiphy [12] shifted its density-stratified error distributions leftward, indicating portability of the image step across pipelines (Fig. S3d).

### 12.4 Head-to-head evaluations showed that SegDecon consistently surpassed StarDist and Spotiphy across density strata and datasets

**Fig. 3:**
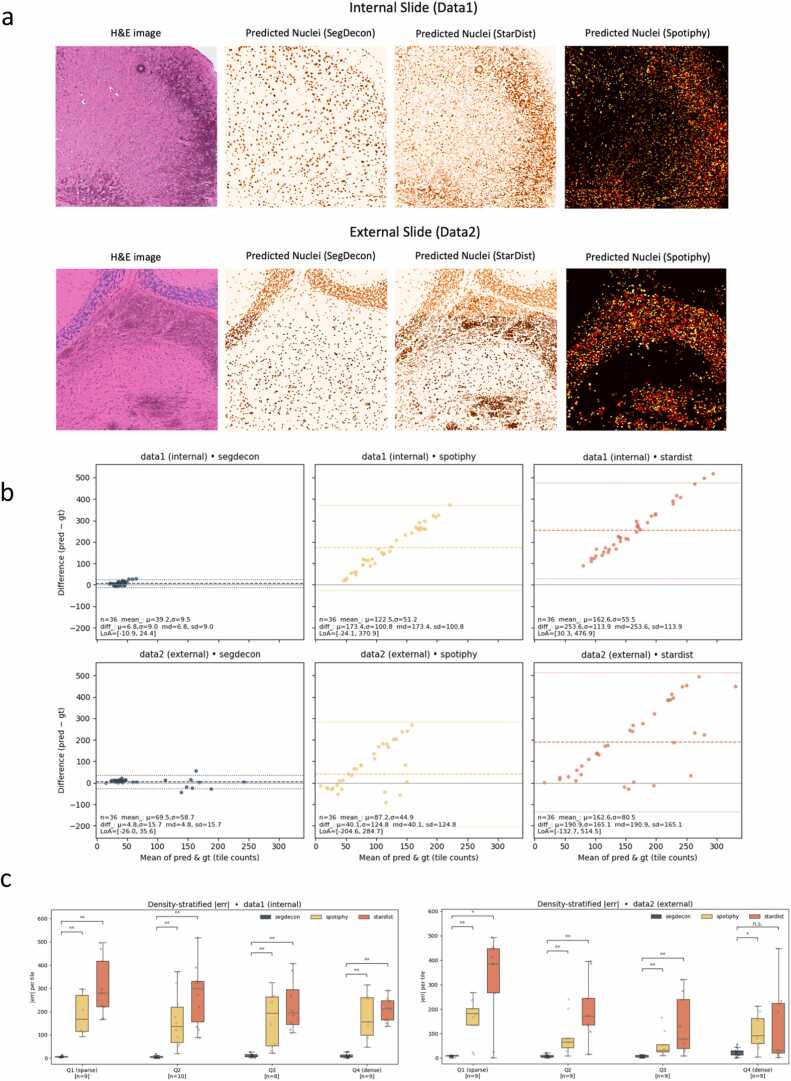
Segmentation performance comparison(a)Internal (Data1) and external (Data2) slides: original H&E, and predicted nuclei from SegDecon, StarDist, and Spotiphy (blue = nuclei; dark brown = artifacts in H&E). (b)Bland-Altman scatter by dataset (rows) and method (columns). Points are tiles; dashed line = mean bias; dotted lines = ±1.96 SD (LoA). Tighter, centered clouds indicate smaller bias and better agreement. Here, gt denotes manual ground-truth (GT) nuclei counts per tile obtained by centroid annotation on the benchmarking tiles; see Methods (‘Manual ground-truth (GT) nuclei annotation and metrics’)(c)Density-stratified tile-wise absolute counting error. Tiles are binned by per-slide ground-truth quartiles (Q1-Q4; Q1 = sparse, Q4 = dense). Within each stratum, methods (SegDecon / Spotiphy / StarDist) are compared via median and IQR; jittered dots are tiles. Brackets show paired Wilcoxon tests with Holm adjustment. Internal and external slides are shown separately.

Across matched tiles from the internal Visium HD-FFPE and external CytAssist fresh-frozen slides, SegDecon outperformed StarDist [8], [9], [10] and Spotiphy [12] in tile-wise nuclei counting across all ground-truth density quartiles (Q1-Q4), with lower medians and narrower variability in every stratum (Fig. 3c). Head-to-head Bland-Altman comparisons showed biases closer to zero and tighter dispersion for SegDecon than for either comparator on both datasets (Fig. 3b). Representative overlays further demonstrated fewer stain-like false positives and less fragmentation, yielding masks that more closely follow nuclear boundaries (Fig. 3a). Taken together, these density-stratified results show consistent, cross-dataset gains over StarDist and Spotiphy without per-dataset tuning (Fig. 3a-c).

### 12.5 Image-informed priors improve deconvolution accuracy and spatial fidelity

**Fig. 4:**
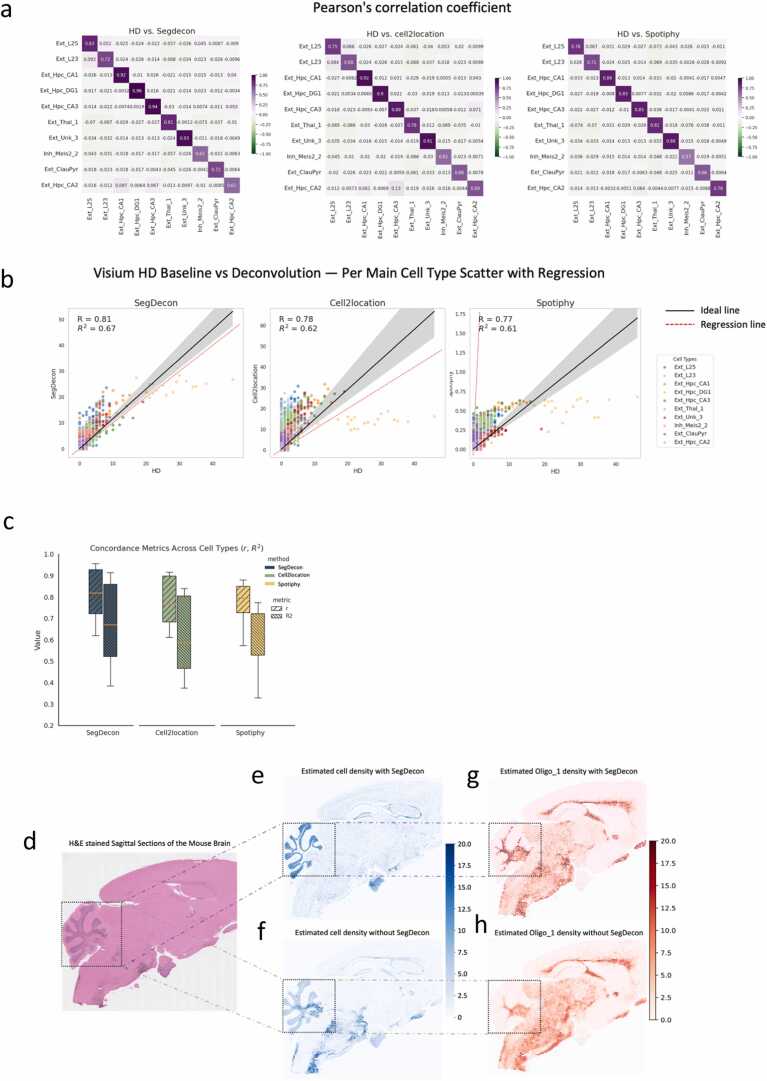
Accuracy evaluation of the deconvolution (a)Heatmaps showing the correlation analysis of deconvoluted cell compositions from SegDecon, cell2location and Spotiphy with HD-ST annotated data. Darker purple shades indicate stronger positive correlations. The top 10 annotated cells were selected. **(b)**Top-K cell types (Data1): per-type scatter with regression; shaded bands indicate the 95 % CI; Scatter plots with regression lines comparing deconvoluted cell compositions from SegDecon, cell2location and Spotiphy with the HD-ST annotated data. Each point represents a specific cell type in a spatial spot, and the shaded area indicates the confidence interval of the regression line. **(c)**Boxplot, Correlation performance across cell types: boxplots of Pearson’s r and R^2^ (SegDecon, cell2location, Spotiphy), showing SegDecon’s higher median and narrower spread.**(d)**Histology image with H&E stain of a sagittal section of the Mouse Brain. **(e)**Estimated cell density with SegDecon. Darker blue indicates higher cell counts per pixel. **(f)**Estimated cell density without SegDecon. Darker blue indicates higher cell counts per pixel. **(g)**Deconvoluted Oligo_1 counts per spot via SegDecon. **(h)**Deconvoluted Oligo_1 distribution via cell2location alone without additional information offered by SegDecon.

Integrating image-derived nuclei information into the deconvolution process markedly enhanced both quantitative accuracy and spatial coherence of inferred cell-type distributions. Across the top annotated populations, SegDecon achieved the highest concordance with HD-ST ground-truth annotations, surpassing cell2location [1] and Spotiphy [12] in per-type correlation heatmaps and aggregate summaries (Fig. 4a-c, g). The improvement was consistent across diverse cellular compositions, indicating that the nuclei-informed priors provide a robust statistical constraint rather than dataset-specific tuning.

Per-type regression analyses further revealed tighter linear fits with narrower confidence intervals, especially for low-abundance or spatially restricted cell types that are typically underrepresented in transcript-based inference (Fig. 4d-f). This pattern suggests that image-informed priors reduce overdispersion in the posterior abundance estimates and stabilize the model’s behavior under sparse gene-expression evidence. By grounding prior strength in observed cellular density, SegDecon maintains flexibility across expression regimes while discouraging biologically implausible fluctuations driven purely by noise or dropout effects.

Spatial reconstructions provided complementary anatomical validation. SegDecon maps more faithfully reproduced tissue microarchitecture, showing crisper, region-consistent cell-type boundaries. For example, Oligo_1 enrichment confined to expected white-matter-like regions, whereas baseline models yielded blurred or misplaced localizations (Fig. 4h). Incorporating ROI-specific nuclei-count moments for prior calibration yielded further gains over global priors, particularly in slides with pronounced heterogeneity (Fig. S2b-d). Together, these results demonstrate that embedding histology-derived priors into Bayesian deconvolution not only improves numerical agreement with high-resolution references but also restores biologically meaningful spatial fidelity without the need for dataset-specific tuning (Fig. 4a-h; Fig. S2).

## 13 Discussion

SegDecon advances spatial transcriptomics by pairing a minimal, transparent image stack with empirically calibrated priors in a deconvolution model, thereby injecting histological evidence of local cellularity without altering transcript counts or the likelihood. Conceptually, the pipeline separates where image information belongs (stabilizing nuclei detection and calibrating total-abundance priors) from where it does not (the observation model for gene counts), which helps preserve interpretability and reduces the risk of data leakage.

Two design choices drive the observed behavior. First, hue-centered preprocessing suppresses stain-related background while preserving chromatic contrast at nuclear borders, which makes instance segmentation less sensitive to slide-specific artifacts and tissue preparation differences. Second, a shared Gamma prior on total abundance, parameterized by (κ, θ) via moment matching to the nuclei-count mean and variance (VMR), aligns prior strength with empirically observed variability. When cellularity varies modestly (low VMR), κ is larger and the prior stabilizes totals; when heterogeneity is high, κ shrinks and the model defers to the data. In practice, this variance control curbs implausible swings in total scale and particularly benefits low-abundance or spatially confined populations.

The evidence aligns with this mechanism. Across datasets and density strata, SegDecon shows lower median error and tighter dispersion in nuclei counting, and higher concordance with HD-ST references in deconvolution, with the largest gains for rare or region-restricted types. Importantly, applying the same HSV preprocessing upstream of Spotiphy [12] shifts its error distributions favorably, indicating that a substantial portion of robustness derives from lightweight color-space denoising rather than pipeline-specific modeling. ROI-specific calibration of the prior yields additional improvements in heterogeneous regions, suggesting that locally adapted prior strength is useful when tissue architecture is mosaic.

The approach is also pragmatic. HSV preprocessing is deterministic and CPU-friendly; segmentation and deconvolution run in minutes on commodity hardware. Defaults are resolution-aware with a small number of interpretable knobs, which supports reproducibility and lowers the barrier to adoption. Because the empirical prior targets only the total abundance term, it sharpens spatial maps and improves alignment with anatomical expectations without directly imposing image features on cell-type proportions.

Limitations remain. Extremely atypical stains, scanner artifacts outside the HSV stack’s operating regime, or large domain shifts in tissue type may still challenge both segmentation and prior calibration. Our evaluations rely on tile-level counts and representative overlays; broader instance-level ground truth would enable finer assessment of boundary fidelity and rare-cell behavior. Finally, a single global prior can be suboptimal in highly mosaic tissues; although ROI-specific calibration helps, hierarchical or smoothly varying hyperpriors may capture local structure more faithfully.

Future work can therefore focus on (i) integrating stain normalization and domain adaptation upstream of segmentation, (ii) learning spatially varying prior strength from multi-slide cohorts, (iii) extending the framework to other imaging and spatial platforms, and (iv) assembling larger, standardized benchmarks with instance-level annotations. Taken together, the results point to a practical principle: modest, well-placed image operations plus empirically anchored priors can materially improve reliability and interpretability in spatial deconvolution, offering a generalizable route for routine analyses without per-dataset tuning.

## CRediT authorship contribution statement

**Yuesi Xi:** Writing – review & editing, Writing – original draft, Visualization, Validation, Software, Methodology, Formal analysis, Conceptualization. **Xun Jiang:** Writing – original draft, Visualization, Validation, Supervision, Methodology, Conceptualization. **Jonas C. Schupp:** Writing – review & editing, Validation, Methodology, Investigation, Funding acquisition. **Cheng-Jian Xu:** Writing – review & editing, Validation, Funding acquisition. **Yang Li:** Writing – review & editing, Validation, Supervision, Resources, Investigation, Funding acquisition, Conceptualization.

## Funding

This project was supported by an ERC Starting Grant 948207 (ModVaccine), the Lower Saxony Center for AI and Causal Methods in Medicine (CAIMed) grant (ZN4257), and the German Federal Ministry of Education and Research (BMBF) grants (01EQ2302A/FEDCOV, 031L0318A/AID-PAIS), the Deutsche Forschungsgemeinschaft (DFG, German Research Foundation) - RTG 2978 - Research Training Group “Understanding and Exploiting Adaptation to Therapy in Gastrointestinal Cancer”, and the Deutsche Forschungsgemeinschaft (DFG, German Research Foundation) under Germany's Excellence Strategy - EXC 2155 - project number 390874280 to Y.L.

## Declaration of Competing Interest

The authors declare that they have no known competing financial interests or personal relationships that could have appeared to influence the work reported in this paper.

## Data Availability

SegDecon was implemented in Python 3.9.18 and is fully documented. The source code, including detailed instructions, example workflows, and pretrained models, is available at: https://github.com/CiiM-Bioinformatics-group/SegDecon.

## Appendix A Supplementary material

Supplementary material Figures 1–3 are provided in Supplementary File 1.Supplementary Methods (including the Parameter Table and Code Listings) and Supplementary Tables are provided in Supplementary File 1
