# Supplementary material for: SegDecon bridges histology and transcriptomics through AI-based nuclei segmentation and image-informed spatial deconvolution
Source: Comput Struct Biotechnol J. 2025 Oct 24;27:4586–96. doi: 10.1016/j.csbj.2025.10.041 (PMC12605641; doi:10.1016/j.csbj.2025.10.041)
Supplement: Supplementary file 1 — Supplementary material Figures 1–3 are provided in Supplementary File 1.Supplementary Methods (including the Parameter Table and Code Listings) and Supplementary Tables are provided in Supplementary File 1 [file mmc1.pdf]

# Supplementary


***Supplementary Figures and legends*** ......2

***Supplementary Methods*** ......7

**1. Data Registration Notes: Visium spot geometry and the effective capture radius** ...... **7**

**2. SegDecon image preprocessing parameters: defaults and roles** ...... **7**

Parameter table (defaults and roles) ...... 8

**3. ROI selection, tiling, and coordinates** ...... **10**

**4. HASS (Hue-Artifact Severity Score): definition and computation** ...... **10**

**5. Manual ground truth (GT) nuclei counts and evaluation metrics** ...... **11**

**6. Segmentation pipelines and controls** ...... **11**

**7. Spatial mapping from polygons to spots and image-derived priors** ...... **12**

**8. Deconvolution model details and prior derivation** ...... **12**

**9. Segmentation evaluation protocol (tile-wise)** ...... **13**

**10. Module-wise ablations of the preprocessing stack (AB01–AB04)** ...... **13**

**11. Control analysis: Spotiphy + HSV** ...... **13**

**12. Runtime, software environment, and seeds** ...... **14**

**13. Code Listings and Evaluation Protocols (Listings S1–S8)** ...... **14**

***Supplementary Tables (schemas & repository location)*** ......16

***Reference*** ......18

## Supplementary Figures and legends

# Fig S.1

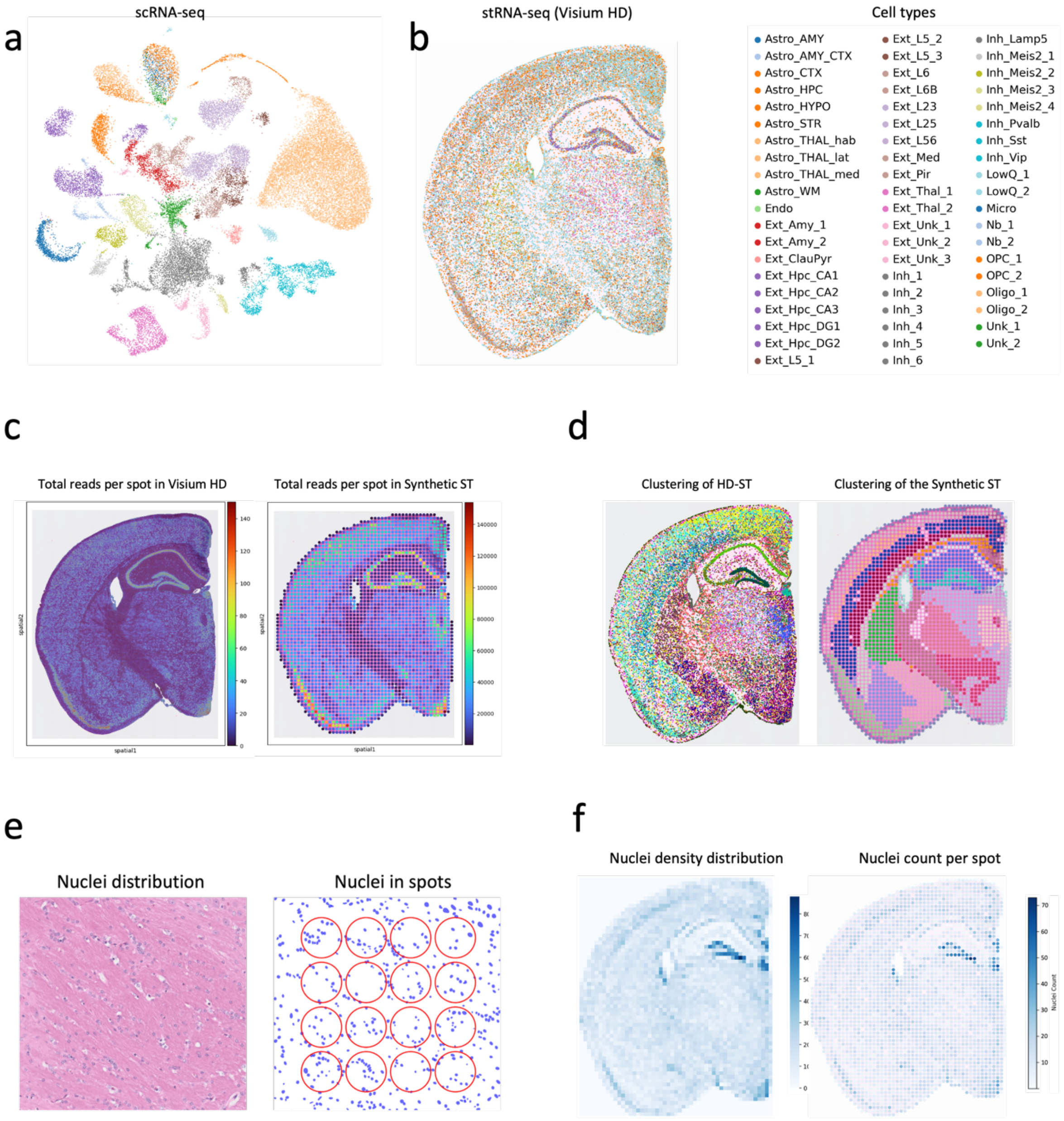

# Fig S.2

a

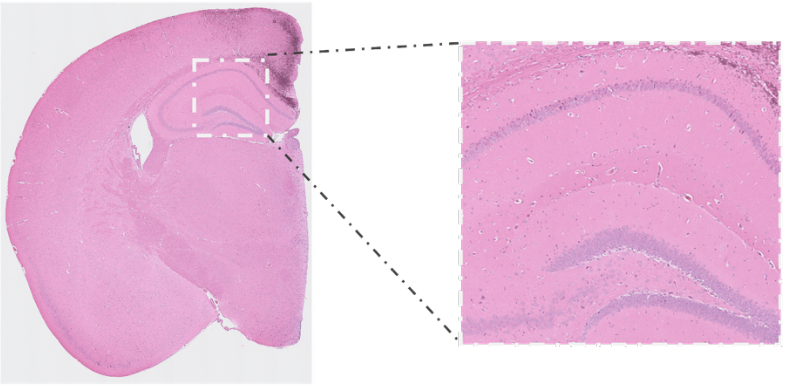

b

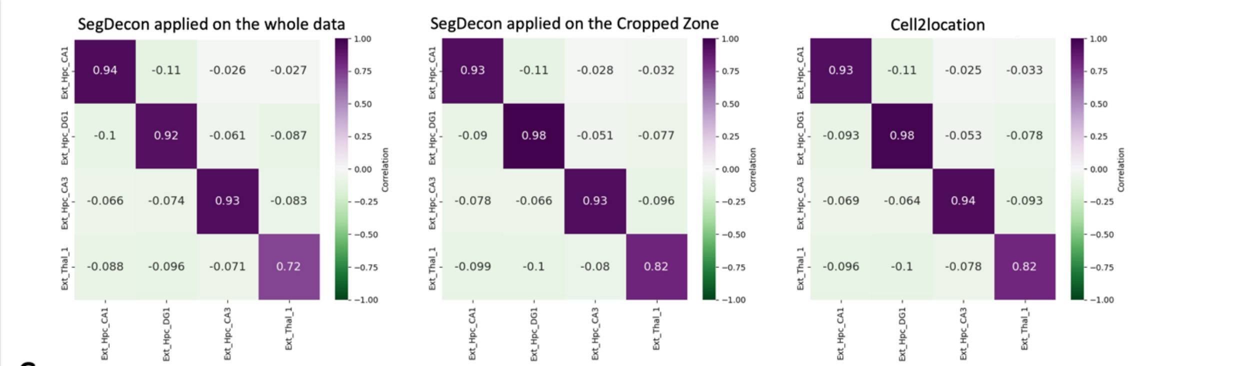
SegDecon applied on the whole data
SegDecon applied on the Cropped Zone
Cell2location
Ext_Hpc_CA1
Ext_Hpc_DG1
Ext_Hpc_CA3
Ext_Thal_1
Correlation
0.94 -0.11 -0.026 -0.027
-0.1 0.92 -0.061 -0.087
-0.066 -0.074 0.93 -0.083
-0.088 -0.096 -0.071 0.72
0.93 -0.11 -0.028 -0.032
-0.09 0.98 -0.051 -0.077
-0.078 -0.066 0.93 -0.096
-0.099 -0.1 -0.08 0.82
0.93 -0.11 -0.025 -0.033
-0.093 0.98 -0.053 -0.078
-0.069 -0.064 0.94 -0.093
-0.096 -0.1 -0.078 0.82

c

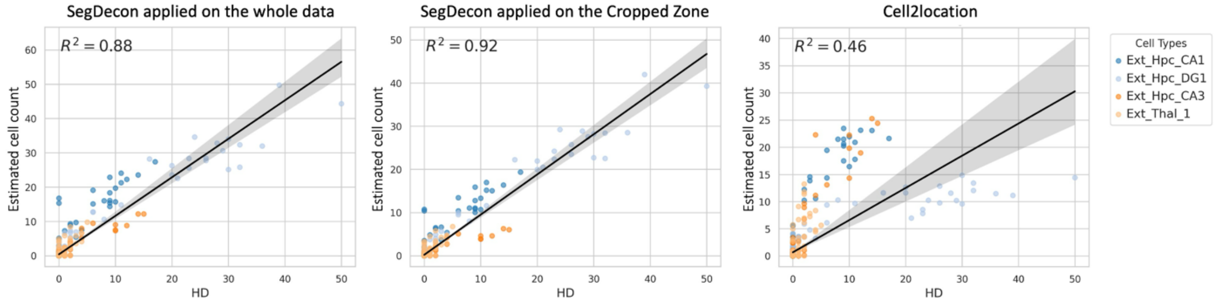
SegDecon applied on the whole data
$R^2 = 0.88$
SegDecon applied on the Cropped Zone
$R^2 = 0.92$
Cell2location
$R^2 = 0.46$
Estimated cell count
HD
Cell Types
Ext_Hpc_CA1
Ext_Hpc_DG1
Ext_Hpc_CA3
Ext_Thal_1

# Fig S.3

a

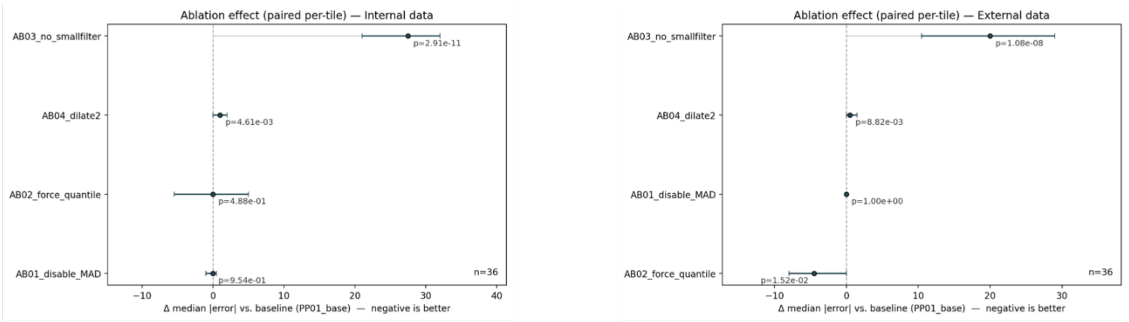
Ablation effect (paired per-tile) — Internal data
AB03_no_smallfilter
AB04_dilate2
AB02_force_quantile
AB01_disable_MAD
p=2.91e-11
p=4.61e-03
p=4.88e-01
p=9.54e-01
n=36
Δ median |error| vs. baseline (PP01_base) — negative is better
Ablation effect (paired per-tile) — External data
AB03_no_smallfilter
AB04_dilate2
AB01_disable_MAD
AB02_force_quantile
p=1.08e-08
p=8.82e-03
p=1.00e+00
p=1.52e-02
n=36
Δ median |error| vs. baseline (PP01_base) — negative is better

b

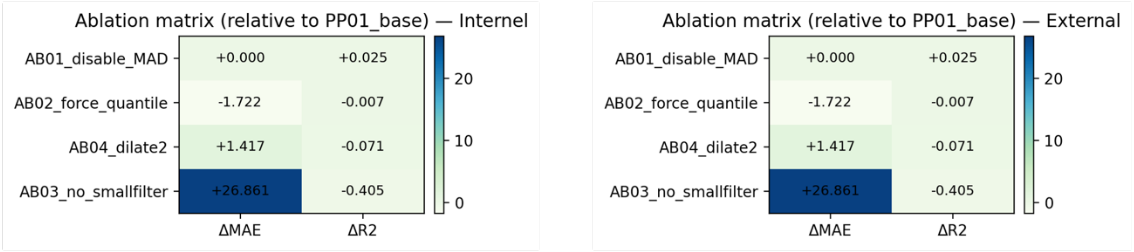
Ablation matrix (relative to PP01_base) — Internel
AB01_disable_MAD +0.000 +0.025
AB02_force_quantile -1.722 -0.007
AB04_dilate2 +1.417 -0.071
AB03_no_smallfilter +26.861 -0.405
ΔMAE
ΔR2
Ablation matrix (relative to PP01_base) — External
AB01_disable_MAD +0.000 +0.025
AB02_force_quantile -1.722 -0.007
AB04_dilate2 +1.417 -0.071
AB03_no_smallfilter +26.861 -0.405
ΔMAE
ΔR2

c

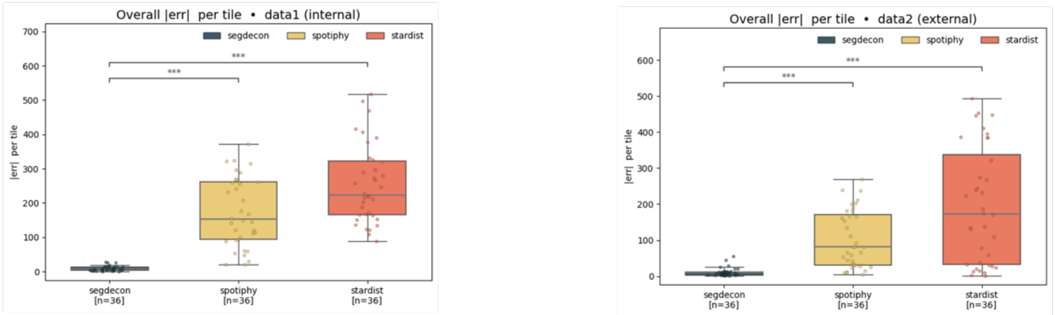
Overall |err| per tile • data1 (internal)
segdecon
spotiphy
stardist
***
|err| per tile
segdecon [n=36]
spotiphy [n=36]
stardist [n=36]
Overall |err| per tile • data2 (external)
segdecon
spotiphy
stardist
***
|err| per tile
segdecon [n=36]
spotiphy [n=36]
stardist [n=36]

d

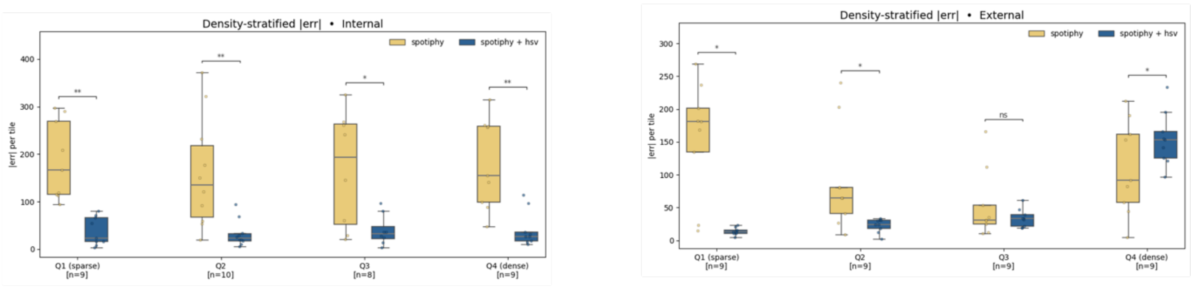
Density-stratified |err| • Internal
spotiphy
spotiphy + hsv
|err| per tile
Q1 (sparse) [n=9]
Q2 [n=10]
Q3 [n=8]
Q4 (dense) [n=9]
Density-stratified |err| • External
spotiphy
spotiphy + hsv
|err| per tile
ns
Q1 (sparse) [n=9]
Q2 [n=9]
Q3 [n=9]
Q4 (dense) [n=9]

**Supplementary figure 1** | Data curation, annotations, and synthetic ST

(a) Consistent cell-type annotation of scRNA-seq Data. A total of 59 distinct cell types were identified and annotated. For clarity, closely related cell types were labeled with the same colors.

(b) stRNA-seq (Visium HD) Data. A total of 59 distinct cell types were identified and annotated. For clarity, closely related cell types were labeled with the same colors.

(c) Total reads of the HD-ST and synthetic data. Heatmaps showing the total reads per spot in the original Visium HD data (left) and the downsampled synthetic ST data (right).

(d) Visualization of unsupervised Leiden clustering applied to the HD-ST data (left) and the synthetic ST data (right).

(e) The left panel shows the distribution of nuclei in the H&E-stained image as an example. The right panel displays nuclei located within virtual spots (red circles), corresponding to the ST data.

(f) The left panel illustrates the nuclei density, representing the total nuclei count within each 100 μm × 100 μm square. The right panel visualizes the nuclei count per spot in (e).

**Supplementary figure 2** | Local-region evaluation of deconvolution

(a) A smaller region with a complex cell distribution was chosen for SegDecon accuracy evaluation.

(b) Correlation of SegDecon-deconvoluted cell compositions. Using hyperparameters from the entire image (left panel), cropped region (middle panel) with HD-ST annotated data. and Correlation of cell2location-based deconvolution results (using hyperparameters from the entire image) with HD-ST annotated data.

(c) Scatter plots with regression; shaded bands indicate the 95% CI; Using hyperparameters from the entire image (left panel), cropped region (middle panel) with HD-ST annotated data. and Correlation of cell2location-based deconvolution results (using hyperparameters from the entire image) with HD-ST annotated data. Each point represents a specific cell type in a spatial spot, and the shaded area indicates the confidence interval of the regression line.

**Supplementary figure 3** | Module-wise ablations and additional controls

(a) Cleveland-dot summary of single-module ablations vs. the default preprocessing (PP01_base). For each ablation, Δmedian|err| is reported per tile (negative = improvement). Paired Wilcoxon p-values are shown (Holm adjusted where applicable). Internal and external slides are plotted separately.

(b) Heatmaps (Internal/External) showing the effect of toggling one preprocessing change at a time (AB01_disable_MAD, AB02_force_quantile, AB03_no_smallfilter, AB04_dilate2). Cells report ΔMAE = MAE (cfg) – MAE (base) (counts/tile; negative = better) and $\Delta R^2 = R^2(\text{cfg}) - R^2(\text{base})$ (positive = better). Across datasets, removing the small-object filter (AB03) yields the largest degradation ($\Delta\text{MAE} \gg 0$ with $\Delta R^2 < 0$); quantile fallback (AB02) slightly reduces MAE with minimal $R^2$ impact; disabling MAD (AB01) is neutral; dilation=2 (AB04) mildly regresses performance.

(c) Overall, per-tile error distributions by slide and method. Boxplots show median and IQR; jittered points are tiles.

(d) Density-stratified errors: Spotiphy vs. Spotiphy+HSV. Tiles are binned by per-slide ground-truth quartiles (Q1–Q4). Within each stratum, |pred−gt| is compared; brackets indicate paired Wilcoxon significance with star notation (*, **, ***) and "ns" for non-significant. Internal and external panels are shown separately.

## Supplementary Methods

### 1. Data Registration Notes: Visium spot geometry and the effective capture radius

**Geometry**. On 10x Visium slides, capture spots have a 55 µm diameter arranged on a 100 µm pitch grid. Vendor scale factors map barcode coordinates into the histology image coordinate system.

**Rationale for an effective capture radius.** For image-side nuclei counting only, each spot is modeled as a disc of radius 45 µm centered at the barcode. This (i) accommodates a proximal not perfectly hard-edged capture footprint, (ii) absorbs small registration errors between the H&E image and barcode coordinates, and (iii) preserves non-overlap because the pitch is 100 µm. The 45 µm value is a single default used across all experiments (no per-dataset tuning).

**Scope.** This affects only image-derived nuclei counts $n_s$ used to compute slide/ROI-level moments$(\overline{n}, \sigma_n^2, VMR)$ for the shared Gamma prior on $m_s$in deconvolution. It does not modify Space Ranger transcript counts or the NB likelihood.

**Intersection rule.** A nucleus counts for a spot if its polygon has positive-area overlap with the 45 µm disc; ties are broken by larger overlap area. Post-hoc polygon area filter: (50, 2000) µm²[1, 2].

**Coordinates.** All geometry is computed in micrometres via the slide's µm/px scale; barcode positions are transformed to the image frame before intersection.

### 2. SegDecon image preprocessing parameters: defaults and roles

This section lists all tunable parameters in the HSV-based histology pipeline with resolution-aware defaults (identical to the released CLI). Use this as the authoritative reference for Methods and for reproducing Figs. 2 & 4.

**Defaults used in all external validations (no per-dataset tuning):**

- Acquisition / expected size: um_per_px = 0.5, nucleus_diam_um = 10
- Tissue mask: S-channel (or hematoxylin via HED), min_tissue_obj_um2 = 400, closing radius derived from nucleus_diam_um
- Hue artifact suppression: percentile clip (5, 95); target artifact area fraction [0.005, 0.30]; quantile fallback if auto thresholds miss target
- Tiny-component removal: min_area_factor = 0.35 of expected nuclear area (optionally override with absolute min_area_um2)
- Background homogenization ("pink band"): q_width = 0.30, k_S = k_V = 2.0, nucleus_dilate_px = 0, smoothing restricted to replaced pixels
- Segmentation: StarDist (pretrained 2D)

- Post-hoc area filter: (50, 2000) µm²

**Presets for atypical slides (optional):**

- Aggressive artifact removal: `--target_area_range 0.01 0.40`, `--min_area_factor 0.4`
- Conservative texture preservation: `--target_area_range 0.005 0.20`, `--min_area_factor 0.2`, `--nucleus_dilate_px 1`

## Parameter table (defaults and roles)

| Parameter (API) | CLI flag | Default | Typical range / options | Role in pipeline |
|---|---|---|---|---|
| `um_per_px` | `--um_per_px` | `0.5` | `0.25–1.0` | Acquisition scale (µm per pixel); drives resolution-aware kernels and area thresholds. |
| `nucleus_diam_um` | `--nucleus_diam_um` | `10.0` | `8–14` | Expected nucleus diameter (µm); sets kernel radius and expected nuclear area. |
| `tissue_method` | `--tissue_method` | `S` | `S` (HSV saturation) or `hematoxylin` | Channel used to obtain initial tissue mask before morphology. |
| `min_tissue_obj_um2` | `--min_tissue_obj_um2` | `400.0` | `200–1200` | Minimum area (µm²) to keep tissue components; removes small debris/holes. |
| `pclip_low_high` | `--pclip_low_high` | `(5, 95)` | `(2, 98)` to `(10, 90)` | Percentile clipping of hue before thresholding (robust to outliers). |
| `target_area_range` | `--target_area_range` | `(0.005, 0.30)` | see presets below | Target fraction of artifact area (relative to tissue) used to select the threshold/tail; falls back to quantiles if unmet. |

| Parameter (API) | CLI flag | Default | Typical range / options | Role in pipeline |
|---|---|---|---|---|
| min_area_um2 | --min_area_um2 | None | e.g., 25–100 | Absolute tiny-component removal threshold (µm²); if None, min_area_factor is used. |
| min_area_factor | --min_area_factor | 0.35 | 0.2–0.5 | Relative tiny-component removal threshold = factor × expected nuclear area. |
| fill_holes | --fill_holes / --no-fill_holes | True | boolean | Whether to fill small holes at the same area threshold. |
| q_width | --q_width | 0.30 | 0.2–0.5 | Quantile width defining the narrow "pink band" around the S-weighted circular hue mean. |
| k_s | --k_s | 2.0 | 1.5–3.0 | MAD multiplier on saturation in the pink-band constraint. |
| k_v | --k_v | 2.0 | 1.5–3.0 | MAD multiplier on value/brightness in the pink-band constraint. |
| nucleus_dilate_px | --nucleus_dilate_px | 0 | 0–3 | Optional safety dilation (pixels) to avoid modifying near-nuclear pixels. |
| smooth_replaced | --smooth_replaced / --no-smooth_replaced | True | boolean | Apply light bilateral smoothing **only** to replaced background. |
| k_px_for_smooth | --k_px_for_smooth | auto (=adaptive_kernel_px) | 5–15 | Effective radius parameter used by the bilateral filter on replaced regions. |

| Parameter (API) | CLI flag | Default | Typical range / options | Role in pipeline |
|---|---|---|---|---|
| *(StarDist post-filter)* `area_min_um2` | *(not a CLI flag in this script)* | `50` | `30–200` | Minimum polygon area (µm²) to keep after instance segmentation (report in text/Supplement). |
| *(StarDist post-filter)* `area_max_um2` | *(not a CLI flag in this script)* | `2000` | `1000–5000` | Maximum polygon area (µm²) to keep after instance segmentation (report in text/Supplement). |

**3. ROI selection, tiling, and coordinates**

For each slide (internal Visium HD-FFPE, external CytAssist fresh-frozen), select one 3,000 × 3,000 px ROI with high artifact fraction yet adequate tissue coverage using the hue-based mask. Partition each ROI into a 6 × 6 grid of 500 × 500 px tiles (36 tiles/slide; 72 total). Export a tile index CSV with global coordinates:

`tile_id, r, c, x, y, w, h` (image origin at top-left; x→right, y→down).

*path: data/meta/<slide>_tile_index.csv.*

**4. HASS (Hue-Artifact Severity Score): definition and computation**

**Purpose**. Stratify tiles by stain-related artifacts to study robustness.

**Components (computed within tissue):**

1. SAF — speckle area fraction;
2. P95_norm — 95th percentile of connected-component size (normalized by tissue area);
3. EOI — edge-overlap index with V-channel Canny edges;
4. BPI — blue/purple nuclei proximity;
5. TDI — local texture degradation index.

**Scoring and binning**. Standardize components within slide; HASS is the unweighted mean of component z-scores. Rank tiles and bin into five within-slide quintiles (levels 1–5, higher=worse). These levels drive Fig. 2d (heatmap) and Fig. 2e (error–noise curves).

### 5. Manual ground truth (GT) nuclei counts and evaluation metrics

**GT definition.** GT comprises manual per-tile nuclei counts used only for evaluation. Annotators clicked nuclei centers on the same 6×6 tiles; the GUI recorded tile-local and full-image coordinates and wrote per-tile counts (units: nuclei/tile).

**Prediction filtering and mapping**. Predicted nuclei are StarDist polygons filtered to (50, 2000) $\mu m^2$ and assigned to tiles by centroid (boundary tolerance $\epsilon 10^{-8}$).

**Metrics**. Tile-wise absolute counting error $|pred - gt|$ (nuclei/tile); Bland–Altman difference vs mean with mean bias and 95% limits of agreement (LoA); paired Wilcoxon tests with Holm adjustment for per-stratum comparisons.

***Outputs.***

meta/<slide>_points_fullimage.csv (one row per clicked nucleus; includes tile_id, x_full, y_full),

meta/<slide>_tile_counts.csv (one row per tile; includes count_gt).

*Path:*

data/meta/<slide>_points_fullimage.csv

data/meta/<slide>_tile_counts.csv

### 6. Segmentation pipelines and controls

We evaluate four segmentation settings under the same post-hoc area filter and tile-mapping rules:

1. SegDecon — HSV preprocessing + StarDist (default);
2. StarDist-only — identical StarDist with HSV off (baseline);
3. Spotiphy — the authors' pipeline (default settings used in paper);
4. Spotiphy+HSV — identical to (3) but prepended with the same HSV preprocessing used by SegDecon (control to isolate color-space denoising effects).

*Path: SegDecon/scripts/eval/ S3–S6.*

**7. Spatial mapping from polygons to spots and image-derived priors**

Nuclei to spot mapping. Map instance-segmented nuclei polygons to 45 µm discs centered at barcode positions (µm units via the slide's scale). Count a nucleus for a spot if there is positive-area intersection; ties resolved by larger overlap area. Obtain per-spot nuclei counts $n_s$.

**Moments and prior.** Across spots $S$:

$$\bar{n} = \frac{1}{|S|}\sum_{s \in S} n_s\,, \qquad \sigma^2 = \frac{1}{|S|-1}\sum_{s \in S}(n_s - \bar{n})^2\,, \qquad VMR = \frac{\sigma_n^2}{\bar{n}}$$

These moments parameterize a shared Gamma prior on the spot-level total abundance $m_s$ (shape-rate form; see §8), used in deconvolution without altering the NB likelihood or transcript counts.

**8. Deconvolution model details and prior derivation**

Likelihood. For gene $g$ at spot $s$, $y_{gs} \sim NB\left(\mu_{gs}, \alpha_g\right)$, with

$$\mu_{gs} = m_s \cdot \sum\nolimits_k w_{ks} r_{gk}$$

*where*: $m_s$ is the total cell abundance at spot $s$; $w_{ks} \geq 0$ with $\sum_k w_{ks}$ = 1 are cell-type proportions with $\sum_k w_{k,s} = 1$, and $r_{gk}$ is the scRNA-seq reference expression of gene $g$ in type k.

Prior on $m_s$. We place a shared Gamma prior $m_s \sim Gamma(\kappa, \theta)$ (shape–rate), obtained by moment matching to slide/ROI nuclei-count moments:

$$\kappa = \frac{\overline{n}}{VMR}, \qquad \theta = \frac{1}{VMR}$$

Lower VMR implies a tighter prior (larger $k$); higher VMR weakens the prior. In the regional analysis (Supplementary Fig. S2), moments are computed on the complex ROI instead of slide-wide.

**Aggregation for Fig. 3**. Correlations are computed per cell type across spots (Top-10 annotated types). Summary statistics aggregate across types to avoid mixing within-type variation with between-type mean/scale differences.

Details in Table_S5_deconv_correlations.csv — Deconvolution correlations

**9. Segmentation evaluation protocol (tile-wise)**

**Common protocol for all methods (Fig. 2, Fig. 4).**

1. Select the 3,000 × 3,000 px ROI; tile into 36 tiles (500 × 500 px).
2. Run each pipeline (SegDecon; StarDist-only; Spotiphy; Spotiphy+HSV).
3. Post-filter polygons to (50, 2000) $\mu m^2$; assign to tiles by centroid.
4. Compare predicted vs GT counts per tile to compute |pred-gt| (nuclei/tile).
5. Produce Bland–Altman plots (difference vs mean), HASS-stratified error–noise curves (Fig. 2e; levels from Fig. 2d), and density-stratified boxplots (Fig. 4c; tiles binned by per-slide GT quartiles Q1–Q4).
6. Perform paired Wilcoxon tests with Holm adjustment per stratum.

*Path: SegDecon/scripts/eval/ S1–S2, S7–S8*

**10. Module-wise ablations of the preprocessing stack (AB01–AB04)**

We performed per-tile paired comparisons against the default configuration (PP01_base) on both slides.

- AB03 — remove small-object filtering: error increases markedly (internal $p=2.91\times10^{-11}$; external $p=1.08\times10^{-8}$).
- AB04 — add 2-px nucleus dilation: performance worsens (internal $p=4.61\times10^{-3}$; external $p=8.82\times10^{-3}$); keep nucleus_dilate_px=0 by default.
- AB02 — force quantile fallback: no change internally (p=0.49); improves externally ($p=1.52\times10^{-2}$), suggesting better resilience under domain shift.
- AB01 — disable S/V MAD constraints: no material effect (internal p=0.95; external p=1.00).

Effect sizes (Δ median |pred-gt|are shown in Cleveland-dot summaries (Supplementary Fig. S3a–b).

**11. Control analysis: Spotiphy + HSV**

To isolate the contribution of color-space denoising from pipeline wrapping, we prepended SegDecon's HSV step to Spotiphy ("Spotiphy+HSV") and repeated the tile-wise evaluation under identical counting/mapping rules. Spotiphy+HSV reduces absolute errors across multiple density strata/datasets relative to vanilla Spotiphy (Supplementary Fig. S3d), indicating that hue-based artifact suppression explains a substantial share of stability gains in artifact-heavy regions. Because the image pipeline never alters transcript counts, deconvolution outputs are unaffected by this control.

## 12. Runtime, software environment, and seeds

All experiments used the default configuration (no per-dataset tuning).

- Workstation: NVIDIA GPU (Tesla 4, 16GB) and 128 GB RAM. CUDA 12.1
- Preprocessing: CPU-only; ≈ 5 minutes per slide (H&E WSI).
- Segmentation & deconvolution: single NVIDIA GPU; wall-clock comparable to baseline pipelines; scales with spot count and gene panel size.
- Software: Python 3.9.18 with documented dependencies.
- Seeds: unless otherwise specified, seed=0 for evaluation scripts.

*End-to-end requirement (segmentation + deconvolution):
- Sequential execution (our default): peak ≈ 5 GB GPU VRAM and ≈ 55 GB system RAM.
- If run concurrently on the same machine (rare): budget ≈ 7 GB GPU VRAM and ≈ 60 GB system RAM.
*Preprocessing (our HSV pipeline, CPU-only): memory scales with image size; typical peaks are ~0.34-0.50 GB RAM for a 4096×4096 tile and ~2-3 GB RAM for a 10k×10k ROI. Whole-slide processing is performed tile-wise to keep memory well below system RAM.

## 13. Code Listings and Evaluation Protocols (Listings S1–S8)

*https://github.com/CiiM-Bioinformatics-group/SegDecon*.

The path is link to Listing S1-8 in GitHub repo (see "Code & data availability"):

- Listing S1 — ROI detection & tiling (scripts/eval/roi_tiling.py): select 3,000×3,000 px ROI; write 6×6 grid index with global coordinates.
- Listing S2 — Manual GT GUI (notebooks/annot_gui.ipynb): click-to-mark nuclei centers; exports per-tile counts and per-point coordinates.
- Listing S3 — SegDecon CLI (scripts/eval/run_segdecon.sh / run_pipeline.py): HSV preprocessing + StarDist; outputs polygons and QC summaries.
- Listing S4 — StarDist-only baseline (scripts/eval/run_stardist_baseline.sh): identical post-filtering but HSV disabled.
- Listing S5 — Spotiphy native (scripts/eval/run_spotiphy.sh): vanilla settings.
- Listing S6 — Spotiphy+HSV (scripts/eval/run_spotiphy_hsv.sh): prepend SegDecon HSV step, then Spotiphy.
- Listing S7 — Tile mapping & metrics (scripts/eval/tile_metrics.py): assign polygons to tiles, compute per-tile counts, |pred- gt|, BA stats.

- Listing S8 — HASS & stratified analyses (scripts/eval/hass_and_strata.py): compute HASS, bin levels (1–5), plot error–noise curves and density-stratified boxplots; paired Wilcoxon (Holm).

## Supplementary Tables (schemas & repository location)

We provide two classes of CSV files: (i) inputs/ground-truth style metadata under data/meta/, and (ii) derived statistics used in the paper under data/tables/. The full files are hosted in the GitHub repository (and mirrored at the data DOI), while the schemas below ensure interpretability from the PDF alone.

https://github.com/CiiM-Bioinformatics-group/SegDecon.

*The path is link to all table mentioned below in GitHub repo.

A. data/meta/ — Inputs & ground truth

- slide_internal_tile_index.csv — 3,000×3,000 px ROI tiled into 6×6 grid.

Columns: `tile_id, r, c, x, y, w, h` (image origin = top-left; x→right, y→down).

- slide_internal_points_fullimage.csv — Manual nuclei clicks (GT) with global coords.

Columns: `tile_id, pid, x_full, y_full, x_crop, y_crop, x_local, y_local.`

- slide_internal_gt_tile_counts.csv — Per-tile GT counts.

Columns: tile_id, filename, count_gt.

- slide_external_tile_index.csv / slide_external_points_fullimage.csv / slide_external_tile_counts.csv — As above for the external slide.

B. data/tables/ — Derived statistics used in the paper

- Table_S1_tilewise_paired.csv — Paired comparisons (e.g., HSV on/off, Spotiphy vs Spotiphy+HSV).

Columns: `slide_id, tile_id, comparison_label, method_a, method_b, abs_err_a, abs_err_b, delta_abs_err, density_bin(Q1–Q4), gt_count, p_value_pairwise, holm_adjusted_p`.

- Table_S2_bland_altman_summary.csv — BA mean bias and LoA per method/slide.

Columns: `slide_id, method, mean_bias, loa_lower, loa_upper, sd, n_tiles, seed.`

- Table_S3_hass_by_level.csv — HASS ladder (levels 1–5) statistics.

Columns: `filename, count_gt, pred_sd, abs_err_sd, ape_sd, pred_sg, abs_err_sg ape_sg,row, col, SAF, P95_norm, EOI, BPI, TDI, HASS noise_level_5bin, noise_level_fixed5,d_abs_err,d_ape`

- Table_S4_density_quartiles.csv — Density-stratified (Q1–Q4) statistics.

Columns: slide_id, density_bin, method, median_abs_err, IQR_abs_err, n_tiles, wilcoxon_p, holm_adjusted_p.

- Table_S5_deconv_correlations.csv — Deconvolution correlations (Fig. 3a–c).

Columns: cell_type, method, r, R2, n_spots, aggregation_note (Top-10 types; per-type across spots; summary=median/mean).

- Table_S6_qc_thresholds.csv — QC thresholds and inclusion rules.

Columns: threshold_name, value, rationale, applied_to.

- Table_S7_ablations_AB01_AB04.csv — Module ablation deltas and p-values.

Columns: tile_id, filename, count_gt, pred_count, err, abs_err

- Table_S8_spotiphy_vs_spotiphyHSV.csv — Control analysis per density stratum.

Columns: slide_id, density_bin, n_tiles, median_base, median_hsv, delta_median, p_raw, p_holm.

Repository paths: data/meta/ and data/tables/ (see Code & Data Availability for DOI and commit hash).

**Notation Cross-Reference**

- $n_s$: image-derived nuclei count for spot s (nuclei/spot).
- $\bar{n}, \sigma_n^2, VMR$: slide/ROI moments used only to set the shared Gamma prior on $m_s$(no per-spot $n_s$ enters the likelihood).
- $m_s$: spot-level total abundance (NB mean factor).
- $w_{k,s}$: cell-type proportion, $\sum_k w_{k,s} = 1$,.
- $r_{gk}$: scRNA-seq reference expression (gene g, type k).
- Polygon area filter: (50, 2000) µm².
- Mapping radius: 45 µm.
- Tiles per ROI: 36 (6×6 of 500×500 px within 3,000×3,000 px crop).
- GT: manual per-tile nucleus counts (centroid clicks), for evaluation only.

## Reference

1. genomics x. What is the spatial resolution and configuration of the capture area of the Visium v1 Gene Expression Slide? 2018 [Available from: https://kb.10xgenomics.com/hc/en-us/articles/360035487572-What-is-the-spatial-resolution-and-configuration-of-the-capture-area-of-the-Visium-v1-Gene-Expression-Slide?utm_source=chatgpt.com.

2. Ni Z, Prasad A, Chen S, Halberg RB, Arkin LM, Drolet BA, et al. SpotClean adjusts for spot swapping in spatial transcriptomics data. Nat Commun. 2022;13(1):2971.
